# Magnitude of academic failure and its associated factors among university students

**DOI:** 10.1192/j.eurpsy.2026.10892

**Published:** 2026-07-31

**Authors:** S. Bradai, N. Ketata, H. Ben ayed, M. Ben Hamida, M. Kassis, J. Jedidi, S. Yaich

**Affiliations:** 1 Hedi Chaker hospital; 2 Habib bourguiba hospital; 3 faculty of medicine of sfax, sfax, Tunisia

## Abstract

**Introduction:**

Students are expected to spend much of their time on their education and need to graduate with good academic results. Thus, academic failure is one of the most serious problems faced by a university student (US).

**Objectives:**

This study aimed to explore the prevalence of academic failure among US and its associated factors.

**Methods:**

A cross-sectional study was conducted among university students at Sfax University, located in southern Tunisia, during the 2024–2025 academic year. A stratified three-stage cluster sampling method was employed to select a representative sample of US. Data collection was performed using an anonymous self-administered questionnaire. Academic failure was identified by an annual average < 10/20 during the last year.

**Results:**

A total of 1071 participants were included in the study, among whom 312 cases were males (29.1%), giving a male to female ratio of 0.41 . The median age of US was 22 [20-23] . There were 1028 US (96%) enrolled in public university and 1037 single US (96.8%). Of all, 61 US (5.7%) were living alone, 171 US (16%) had chronic disease, 160 US (14.9%) practiced physical activity, 920 US (85.9%) spent time on leisure activities and 378 US (35.3%) were frequently meeting their friends. Low monthly income was noted among 135 cases (12.6 %).

The prevalence of academic failure among US was 14% (n=150). Academic failure was statistically associated with male gender (OR=1.125;P=0.046), frequently meeting friends (OR=1.165;P=0.026), Alcohol comsumption (OR=1.133; P<0.001), Tobacco use (OR=1.125; P=0.009) and illicit drug use (OR= 1.035; P=0.049).

**Conclusions:**

Academic failure among US was identified as a major concern, with substance use emerging as the primary associated factor. Early detection of academic difficulties and substance use could be crucial in preventing these issues.

**Disclosure of Interest:**

None Declared

